# Challenges in Regulating Synthetic New Psychoactive Substances: Lessons from Japan’s Generic Scheduling against Hexahydrocannabihexol

**DOI:** 10.31662/jmaj.2024-0110

**Published:** 2024-11-18

**Authors:** Natsuki Yokoyama, Tatsuki Ikejiri, Hayase Hakariya

**Affiliations:** 1 Laboratory for Human Nature, Cultures and Medicine, Kyoto, Japan; 2 Department of Pharmacy, Chubu Tokushukai Hospital, Nakagami, Japan; 3 Interfaculty Institute of Biochemistry, University of Tuebingen, Tuebingen, Germany; 4 Institute for Pharmaceutical and Social Health Sciences, Ise, Japan

**Keywords:** public health, health policy, cannabis, tetrahydrocannabinol, drug regulation, generic scheduling, designer drugs

## Abstract

On December 2, 2023, Japan’s Ministry of Health, Labour and Welfare (MHLW) announced an ordinance regulating the possession, consumption, and distribution of hexahydrocannabihexol (HHCH) except for medical purposes. HHCH, a synthetic cannabinoid, has been linked to central nervous system symptoms, including nausea, dizziness, and numbness, presumably due to its structural similarity to tetrahydrocannabinol. This regulatory action reflects Japan’s historical drug regulation approach, which has evolved to address synthetic substances not covered by earlier laws. The emergence of new psychoactive substances has led to increased poisoning cases and necessitated Japan to introduce a generic scheduling system and collectively regulate these compounds. Despite the reduction in designer drug-related arrests following system implementation, recent trends have shown a resurgence in arrests, partly because of the increased online accessibility of these substances. The persistence of HHCH gummy manufacturers highlights the limitations of current regulations. Thus, enhancing health literacy and social responsibility among consumers and proactive measures by healthcare professionals are essential to mitigate the public health risks associated with these emerging substances. Regulatory frameworks should prioritize public health over economic benefits.

On December 2, 2023, Japan’s Ministry of Health, Labour and Welfare (MHLW) announced the enforcement of an ordinance that regulates the possession, consumption, and distribution (sale, and purchasing) of hexahydrocannabihexol (HHCH) except for medical purposes ^(1)^. HHCH is a synthetic substance that mimics the effects of cannabis. The framework was adopted according to several reported cases in which individuals consumed edible gummy products containing HHCH (HHCH gummy) that caused central nervous system (CNS) symptoms, such as nausea, dizziness, and numbness. Similar edible products containing tetrahydrocannabinol (THC) are sold globally, and their illicitness depends on countries ^(2)^. Symptoms for those who consumed HHCH gummies were presumably because of the chemical structure of HHCH, which is similar to the main component of marijuana, THC, which acts as an agonist of cannabinoid receptors (CB1 and CB2) (Figure 1).

**Figure 1.**
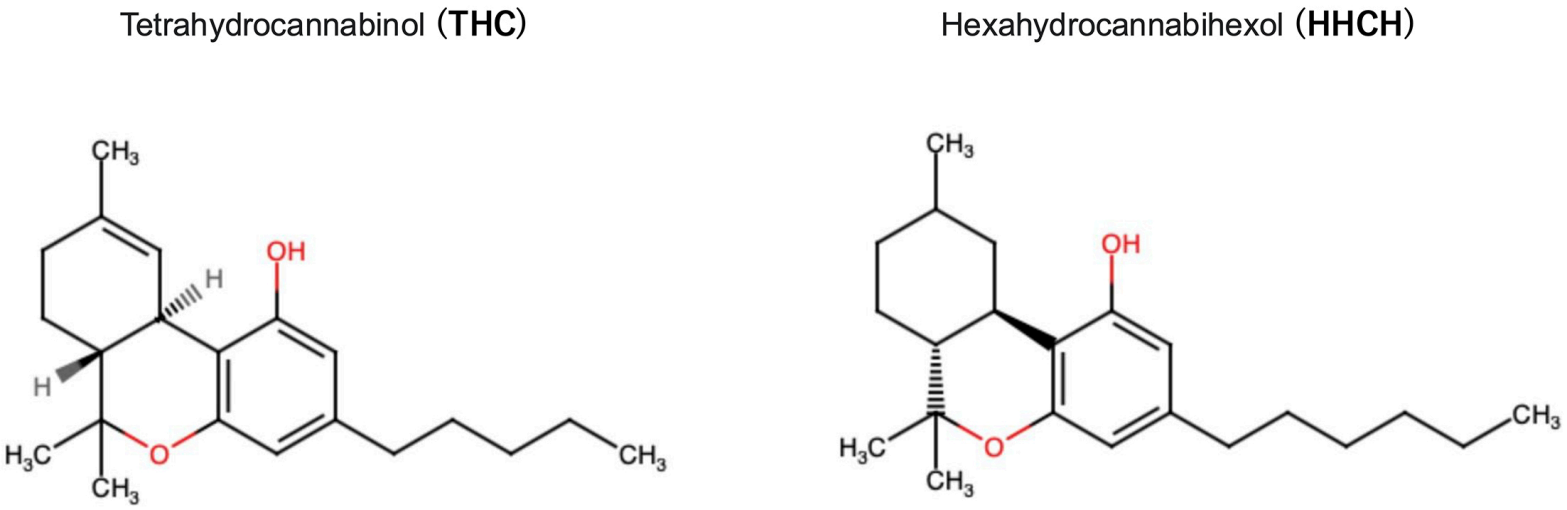
Chemical structures of tetrahydrocannabinol (THC) and hexahydrocannabihexol (HHCH).

This series of events serves as a microcosm of Japan’s drug regulation approach. Historically, Japan has regulated illegal drugs by designating natural cannabis plant (*Cannabis sativa L.*) and products from the plant under the Cannabis Control Act (1948-) and by designating individual chemical structures under the Narcotics and Psychotropics Control Act (1953-), with up to 221 designated cannabis plants as of April 2022. Until now, these laws have been revised and have expanded targets. As a loophole, compounds not specified by these laws are not subject to legal restrictions as illegal drugs. In addition, these regulations are only valid if the components of the products are proven to be medical drugs. However, manufacturers are selling illegal drug products with different labels, such as deodorants, video cleaners, and research reagents, making it difficult to identify medical components. Taking advantage of these traits, synthetic cannabinoids outside these regulations began to be abused domestically in the late 1990s in various forms, such as incense, bath salts, and liquids (later domestically known as designer drugs), which were packaged with counterfeit labels. To address these issues, the MHLW amended the Pharmaceutical Affairs Law (currently known as the Act on Pharmaceutical and Medical Devices) in 2006, allowing a minister to regulate nonmedical substances by designation of individual compounds ^(3)^, known as Designated Substances (currently 2439 substances are controlled as of January 19, 2024).

This designation framework generated a cat-and-mouse scenario because new psychoactive substances, such as synthetic cannabinoids and cathinones, emerged one after another. As a result, the reported number of patients who were transported to emergency facilities because of designer drug poisonings drastically increased in 2012 ^(4)^ and was broadcasted nationwide in the mainstream media. To proactively address the emergence of new psychoactive substances, the MHLW introduced a comprehensive system (generic scheduling) in 2013 ^(5)^, allowing them to collectively regulate potentially hazardous and structurally similar substances based on their chemical backbone structures. This system, which has also been applied in the United Kingdom, initially intended to comprehensively designate naphthoylindole-type synthetic cannabinoids with particular substituents in Japan, resulted in 772 synthetic cannabinoids being designated simultaneously, followed by the designation of 840 cathinone derivatives ^(6)^. Owing to the introduction of the comprehensive regulation framework, the number of designer drug-related arrests reduced drastically year by year ^(6)^. However, since 2022, the number of arrests has been increasing ^(7)^. The escalating arrests may be attributed to increased accessibility to manufactured products, including divergent pharmaceutical substances, in the digital age; indeed, cases in which individuals illegally acquire medications via social networking services have been reported in Japan ^(8), (9)^.

These historical backgrounds indicate the limitations of regulatory approaches despite repeated amendments: individuals can find loopholes and behave differently regarding inappropriate drug use in the swift changes of the era. The most recent amendment reflects the shift of the governing law for THC and related substances from the Cannabis Control Act to the Narcotics and Psychotropic Substances Control Law, which allows the medical use of THC or cannabis-derived substances ^(10)^. Although it remains unclear how these changes will impact the misuse of these drugs, it can be reasonably asserted that regulations are not immutable entities. To prevent the widespread abuse of drugs, dedicated efforts with generic scheduling are indispensable at the very least. Product regulation according to their pharmacological effects instead of chemical structures could also be beneficial, as New Zealand has already installed; the government defines psychoactive substances as anything capable of inducing a psychoactive effect (regardless of their chemical structure) and regulates them unless approved or specially listed as a medical or controlled drugs, precursor substance, herbal remedy, food, dietary supplement, tobacco product, or alcohol ^(11)^.

Beyond regulations, another approach to preventing inappropriate drug or product use would be to form a steady social and ethical basis that could foster health and consumer literacy. Despite their adverse effects on public health, the manufacturers of HHCH gummies have extended their intention to continue to sell them by exploring loopholes in the legal framework. This underscores the insufficiency of regulations based on pre-existing substances. Therefore, consumers should acquire health literacy so they do not obtain and consume potentially harmful items nor interact with dealers of such potentially harmful items. Such consumer abilities are advocated globally, encompass the concept of ethical consumerism, and are outlined by the Consumer Affairs Agency of Japan ^(12), (13)^. Prudent consideration is required regarding how a product affects one’s health and reflects on the social impact of benefiting the manufacturer through its purchase. Comprehensively enriching educational opportunities such as school curricula, private sector initiatives, and government promotion could be effective as a society. In this regard, cases, in which celebrities, i.e., entertainers, famous online influencers, or sports players advertise cannabis edibles or related products should also be questioned. Healthcare professionals may assume social responsibility to collect information on dubious products and conduct awareness campaigns. By recognizing diverse designer drugs and the serious health risks associated with their use, healthcare providers can alert their patients to the harmful effects of these drugs/products and prevent the widespread distribution and abuse of these emerging psychoactive substances.

In conclusion, regardless of the stringency of regulations imposed on manufacturers, eradicating those prioritizing their financial gains over social welfare and public health would be unfeasible, particularly in a capitalist paradigm. Thus, in addition to regulatory structures, implementing social frameworks that prevent such business cultures or practices may be necessary. We advocate that economic benefits should not supersede individual health.

## Article Information

### Conflicts of Interest

None

### Acknowledgement

The authors thank Mrs. M. Hara and Mrs. M. Matsuda for supporting research and maintaining our online laboratory. H.H. is supported by TOYOBO BIOTECHNOLOGY FOUNDATION Fellowship and JSPS Overseas Research Fellowships outside the submitted work.

### Author Contributions

TI and HH conceptualized the work, and collected the information required for the project. NY and TI wrote the original manuscript. HH collected and provided further fruitful information reviewed the original draft critically, and discussed and interpreted the contents. All authors read through the submitted manuscript and approved to be published. Natsuki Yokoyama, Tatsuki Ikejiri and Hayase Hakariya contributed equally to this work.

### ORCID iD

Natsuki Yokoyama: orcid.org/0009-0007-9723-2754

Tatsuki Ikejiri: orcid.org/0009-0006-3624-6135

Hayase Hakariya: orcid.org/0000-0002-9121-4551
